# Supplementary material for: Systematic design and experimental demonstration of bianisotropic metasurfaces for scattering-free manipulation of acoustic wavefronts
Source: Nat Commun. 2018 Apr 9;9:1342. doi: 10.1038/s41467-018-03778-9 (PMC5890261; doi:10.1038/s41467-018-03778-9)
Supplement: Supplementary file 1 — Supplementary Information(PDF 1492 kb) [file 41467_2018_3778_MOESM1_ESM.pdf]

Supplementary material for the paper

"Systematic design and experimental demonstration of bianisotropic metasurfaces for scattering-free manipulation of acoustic wavefronts"

Junfei Li, Chen Shen, Ana Díaz-Rubio, Sergei Tretyakov, and Steven Cummer

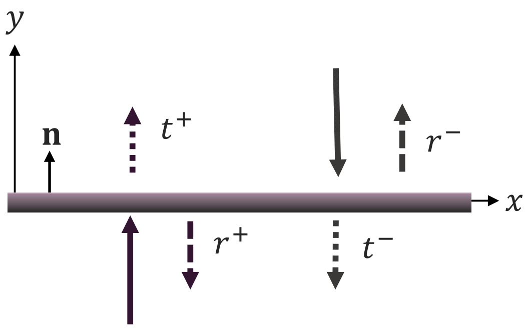

Supplementary Figure 1: Illustration of the forward (+) and backward (-) reflection and transmission coefficients. For a bi-anisotropic unit cell, the forward and backward transmission coefficients are identical, while the reflection phases are different for different directions

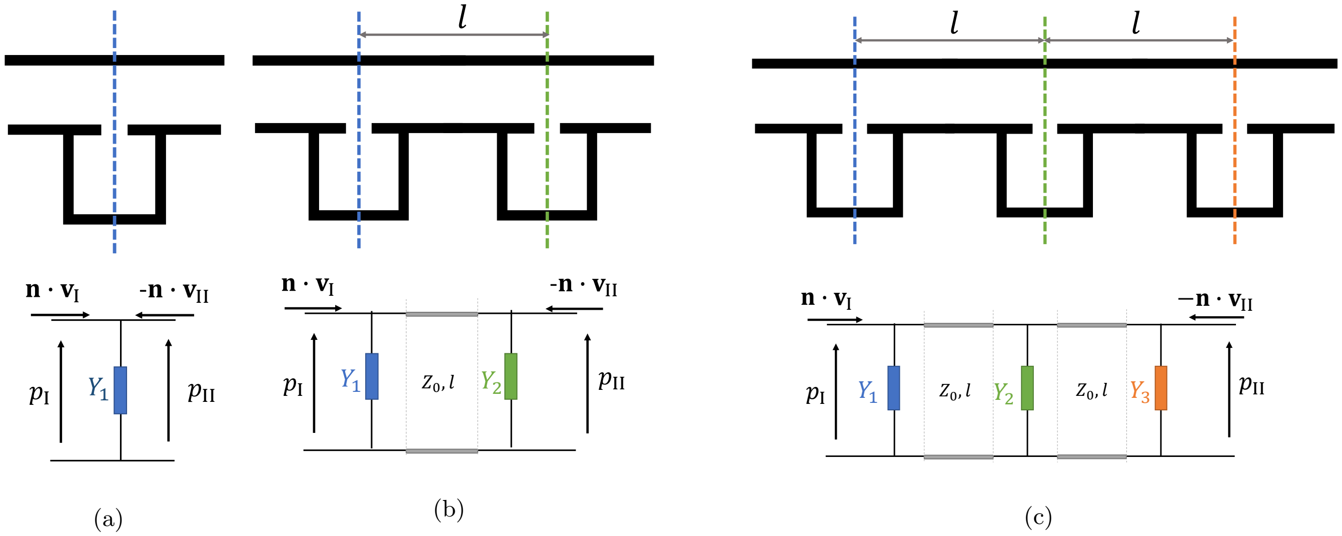

Supplementary Figure 2: Control of the asymmetric response with side-loaded resonators. (a) Single resonators and the corresponding equivalent circuit. (b) Asymmetric cell with two resonators and the equivalent circuit. (c) Asymmetric cell with three resonators and the equivalent circuit.

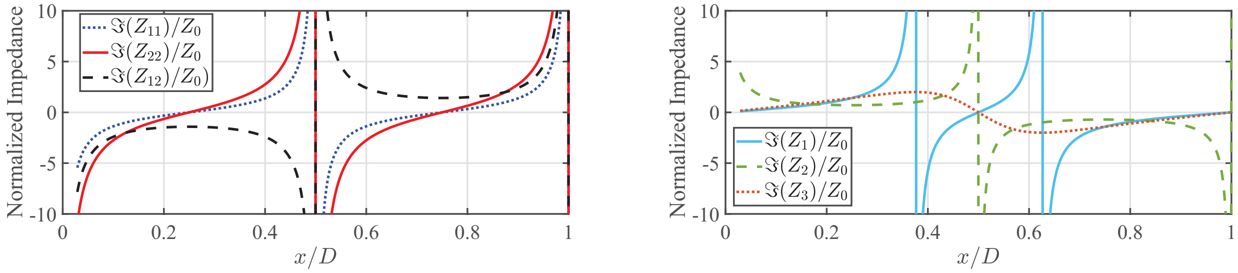

(a) (b)

Supplementary Figure 3: Gradient metasurface for anomalous refraction ($\theta_\mathrm{i} = 0^\circ$ and $\theta_\mathrm{t} = 80^\circ$). (a) Values of the impedance matrix in one period of the metasurface and (b) impedances of the three resonators when $l = \lambda_0/4$ which produce the desired response.

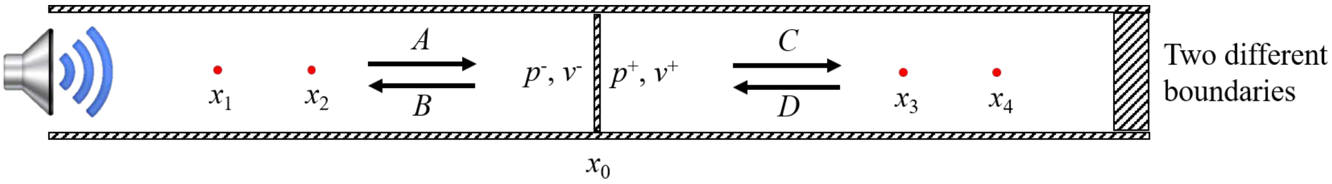

Supplementary Figure 4: Setups of the standard 4-microphone method. x_1, x_2, x_3, x_4 shows the location of the microphones, x_0 is the location of the unit cell. A, B, C, D shows the incoming and outgoing plane waves. Measurements are performed with two different boundaries at the end of the tube.

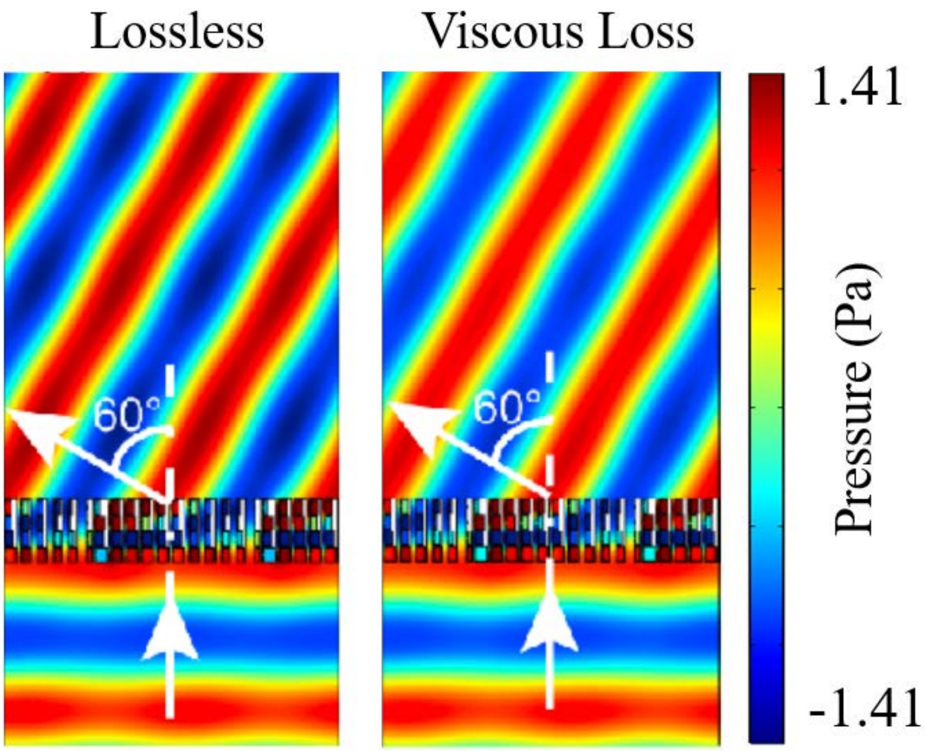

Supplementary Figure 5: Simulated acoustic field (real part) of the designed perfect metasurface for $\theta_\mathrm{i} = 0^\circ$ and $\theta_\mathrm{t} = 60^\circ$ using the proposed non-resonant structures. Lossless simulation (left) and simulation with viscous loss (right) in air.

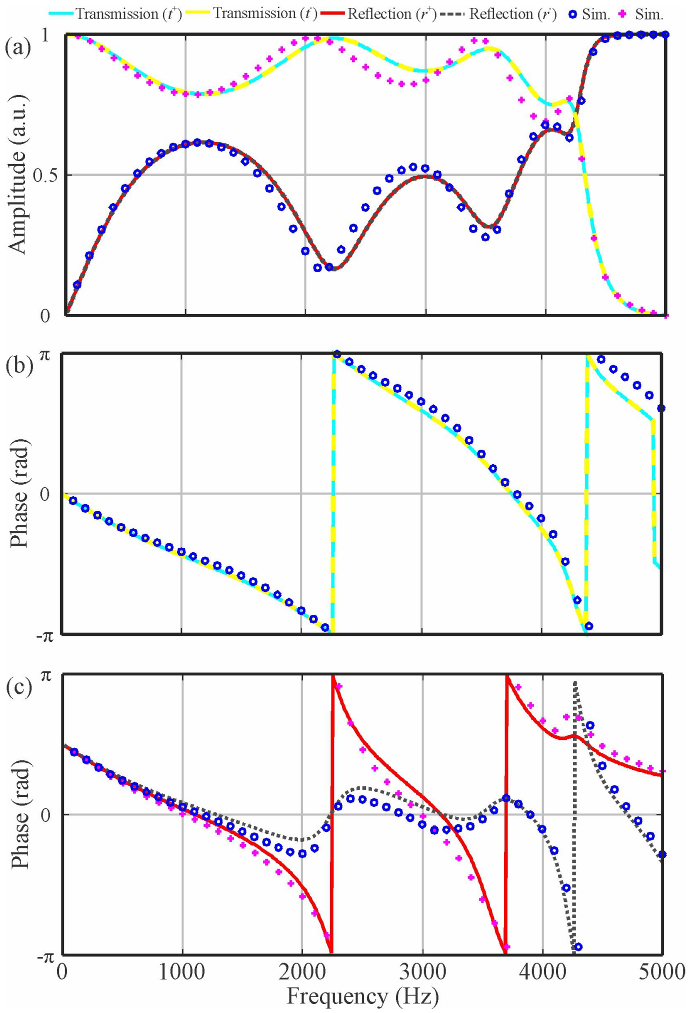

Supplementary Figure 6: Comparison between transfer matrix method and numerical simulations. The parameters are the same as in Fig.2. (a) shows the Amplitudes of the transmission and reflection coefficients; (b) shows the phase of transmission coefficients; (c) shows the phase of reflection coefficients. In a bi-anisotropic unit cell, only reflection phases are different for both directions.

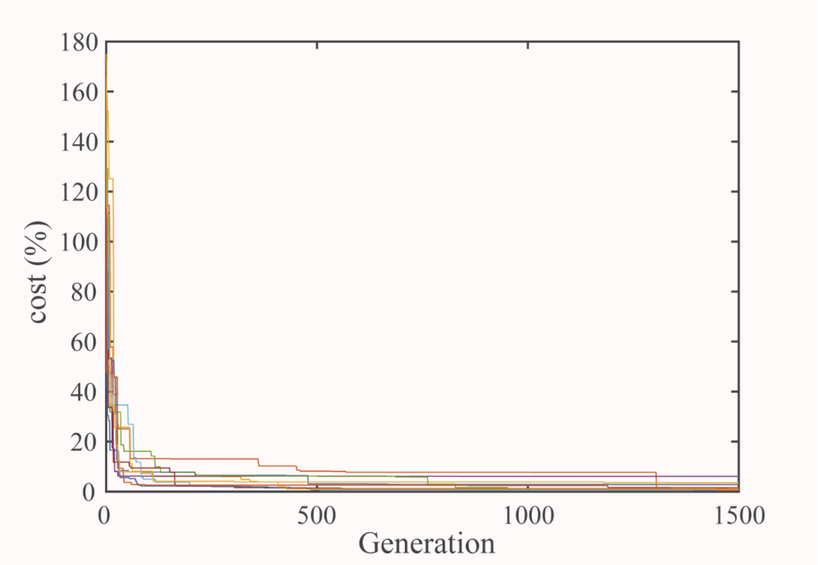

Supplementary Figure 7: The evolution of the cost function with the evolution of the algorithm. The figure shows 10 convergence plots.

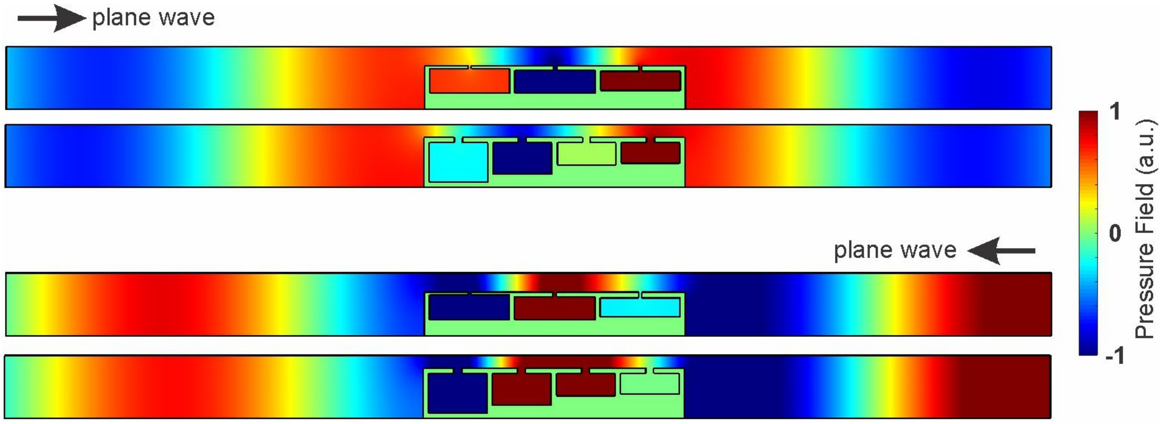

Supplementary Figure 8: Comparison of 3-resonator design and 4-resonator design.

		Resonator A		Resonator B		Resonator C		Resonator D		coefficients		
Cell	$w_1(mm)$	w_a (mm)	f_a (kHz)	w_b (mm)	f_b (kHz)	w_c (mm)	f_c (kHz)	w_d (mm)	f_d (kHz)	r^+	r^-	$t^\pm$
1	2.4	7.6	4.61	6.1	5.21	4.3	6.30	4.0	6.54	-0.24+0.39i	0.46-0.00i	-0.77+0.44i
2	3.2	6.8	4.78	5.3	5.48	5.6	5.32	5.1	5.59	-0.13-0.14i	0.18-0.06i	-0.95-0.25i
3	3.5	6.5	4.86	4.2	6.14	4.1	6.22	4.6	5.86	0.28-0.08i	0.16-0.24i	-0.57-0.77i
4	4.6	5.4	5.24	4.9	5.52	2.8	7.34	4.6	5.71	0.31+0.028i	0.29-0.30i	-0.04-0.91i
5	4.6	5.4	5.24	3.0	7.10	1.0	11.23	1.9	8.78	0.01+0.45i	0.37-0.25i	0.41-0.79i
6	6.8	3.2	6.63	3.1	6.73	0.1	16.20	0.1	16.20	-0.20+0.17i	0.25-0.08i	0.85-0.45i
7	1.4	8.6	4.52	6.1	5.50	6.9	5.13	2.4	8.91	0.68-0.11i	0.18-0.67i	0.48+0.54i
8	1.9	8.1	4.54	7.8	4.64	7.0	4.94	5.6	5.59	0.01-0.11i	0.08-0.08i	0.90+0.42i
9	2.1	7.9	4.56	7.6	4.67	6.5	5.10	5.8	5.43	0.46+0.14i	0.28-0.39i	0.28+0.83i
10	2.1	7.9	4.56	6.8	4.97	4.9	5.95	5.7	5.48	0.37+0.43i	0.47-0.31i	-0.11+0.82i
11	2.2	7.8	4.58	6.6	5.03	3.9	6.69	4.7	6.06	0.04+0.53i	0.52-0.12i	-0.50+0.68i

Supplementary Table 1: Design parameters and resonance frequencies of the individual resonators of the scattering-free bianisotropic metasurface to steer a normal incident wave toward $\theta_\mathrm{t} = 60^\circ$, implemented with 11 cells within one period.

Cell	cost(%)	w (mm)	w_1 mm)	w_a (mm)	w_b (mm)	w_c (mm)	w_d (mm)	r^+	r^-	$t^\pm$
1	17.42	30.4	10.4	9.1	7.5	11.4	10.2	-0.33+0.39i	0.23-0.45i	-0.48+0.72i
2	4.50	30.4	11.9	6.5	16.2	8.6	16.0	-0.04-0.51i	0.39-0.34i	0.79+0.32i
3	1.52	30.4	15.6	9.2	7.2	2.0	3.5	0.07+0.53i	0.38-0.37i	0.27-0.80i
4	5.94	30.4	10.9	10.6	10.6	5.2	1.7	-0.06-0.44i	0.33-0.31i	-0.84-0.30i

Supplementary Table 2: Design parameters of the individual resonators of the scattering-free bianisotropic metasurface to steer a normal incident wave toward $\theta_t = 70^\circ$ implemented with 4 cells within one period.

Cell	cost(%)	w (mm)	w_1 mm)	w_a (mm)	w_b (mm)	w_c (mm)	w_d (mm)	r^+	r^-	$t^{\pm}$
1	4.07	29.0	4.6	9.6	10.7	4.4	4.6	-0.07+0.71i	0.49-0.51i	-0.30+0.64i
2	14.32	29.0	7.1	6.6	19.3	7.4	19.1	-0.15-0.68i	0.53-0.45i	0.68+0.23i
3	15.40	29.0	16.5	3.8	9.9	0.3	6.1	0.33+0.67i	0.57-0.49i	0.13-0.65i
4	9.47	29.0	14.5	7.5	8.7	11.5	9.4	-0.21-0.64i	0.48-0.46i	-0.72-0.18i

Supplementary Table 3: Design parameters of the individual resonators of the scattering-free bianisotropic metasurface to steer a normal incident wave toward $\theta_t = 80^\circ$ implemented with 4 cells within one period.

Supplementary Note 1. Analysis of the scattering properties of lossless and reciprocal metasurfaces

The objective of this supplementary note is to evaluate the required properties of a metasurface for producing a specific scattering response, i.e., a set of transmission and reflection coefficients. The scattering properties of the metasurface are defined through the transmission and reflection coefficients in the forward (+) and a backward (-) direction [see Supplementary Figure 1] which can be expressed in terms of the scattering matrix as:

$$S = \begin{bmatrix} r^+ & t^- \\ t^+ & r^- \end{bmatrix} \tag{1}$$

In our analysis we will consider reciprocal metasurfaces which satisfy $S = S^{\mathrm{t}}$, where the superscript t denotes the transpose of the scattering matrix. This condition can also be written as $t^+ = t^- = t$. In addition, we will work with lossless metasurfaces which have to satisfy $S^* S^{\mathrm{t}} = I$, with the superscript * being the complex conjugate and I being the identity matrix. In other words, a lossless and reciprocal metasurface will satisfy:

$$\begin{bmatrix} |r^+|^2 + |t|^2 & r^{+*}t + t^* r^- \\ t^* r^+ + r^{-*}t & |r^-|^2 + |t|^2 \end{bmatrix} = \begin{bmatrix} 1 & 0 \\ 0 & 1 \end{bmatrix} \tag{2}$$

which can be simply written as

$$|t|^2 + |r^{\pm}|^2 = 1 \tag{3}$$

$$r^+ t^* + t r^{-*} = 0 \tag{4}$$

The complex value of the transmission and reflection coefficients can be expressed as $r^+ = |r^+| e^{j\phi_{\mathrm{r}}^+}$, $r^- = |r^-| e^{j\phi_{\mathrm{r}}^-}$, and $t = |t| e^{j\phi_{\mathrm{t}}}$. From Supplementary Eq. (3), we can see that the amplitudes of the reflection coefficients in forward and backward direction have to be the same, $|r^+| = |r^-| = |r|$. The second

condition for a lossless and reciprocal metasurface shown in Supplementary Eq. (4) can be rewritten as

$$|r||t|e^{j(\phi_{\mathrm{r}}^{+}-\phi_{\mathrm{t}})}+|r||t|e^{-j(\phi_{\mathrm{r}}^{-}-\phi_{\mathrm{t}})}=0 \tag{5}$$

This condition is fulfilled if the phases of the reflection coefficient satisfy

$$(\phi_{\mathrm{r}}^{+}+\phi_{\mathrm{r}}^{-})=2\phi_{\mathrm{t}}+(2n+1)\pi \tag{6}$$

where $n = 0, 1, 2....$ In summary, any lossless and reciprocal metasurface must satisfy Supplementary Eqs. (3) and (6).

For non-bianisotropic metasurfaces, the phases of the reflection coefficient are equal, $\phi_{\mathrm{r}}^{+} = \phi_{\mathrm{r}}^{-}$. In this case, the relation between the magnitudes and the phases of the reflection and transmission coefficients are uniquely determined by Supplementary Eqs. (3) and (6). From a practical point of view, one can fully design (magnitude and phase) the reflection or transmission coefficient but not having simultaneous control of both of them with non-bianisotropic metasurfaces.

On the other hand, bianisotropic metasurfaces generate different phases in the reflection coefficient when the metasurface is illuminated from different sides, $\phi_{\mathrm{r}}^{+} \neq \phi_{\mathrm{r}}^{-}$. In this case, the magnitude of reflection and transmission coefficients will still be related by Supplementary Eq. (3), but two phases can be simultaneously designed at will [the third phase is automatically determined by satisfying Supplementary Eq. 6)].

Supplementary Note 2. Minimum requirements for full control of the bianisotropic response

In this supplementary section, we deeply analyze how to control the bianisotropic response with side-loaded resonators. This analysis will determine the minimum number of resonators that allows full control of the response. We can analyze the response of the resonator as a local impedance which produce continuity of the pressure at both sides of the resonator [see Supplementary Figure 2(a)]. The proposed building element is a symmetric structure and consequently any bianisotropic response can be found. In order to force the asymmetric response of the cell, we can combine the effect of two different resonators separated a distance l. The relation between the pressure and velocity fields at both sides of the cell can be written as

$$\begin{bmatrix} p_{\mathrm{I}} \\ p_{\mathrm{II}} \end{bmatrix} = \begin{bmatrix} \frac{M_{11}}{M_{21}} & \frac{1}{M_{21}} \\ \frac{1}{M_{21}} & \frac{M_{22}}{M_{21}} \end{bmatrix} \begin{bmatrix} \mathbf{n}\cdot\mathbf{v}_{\mathrm{I}} \\ -\mathbf{n}\cdot\mathbf{v}_{\mathrm{II}} \end{bmatrix}. \tag{7}$$

where $M_{11} = \cos(kl) + jY_2\sin(kl)$, $M_{22} = \cos(kl) + jY_1\sin(kl)$, and $M_{21} = [Y_1 + Y_2]\cos(kl) + j[Y_0 + Y_2Y_1Z_0]\sin(kl)$. We can see that this structure allows bianisotropic response if $Y_1 \neq Y_2$. However, for full control of the response we need to include the separation between the resonators as a parameter of the design. This solution is not suitable for gradient metasurfaces, where the thickness of the metasurface has to be constant.

For this reason, we find that the minimum requirement for full control of the scattering properties with a constant thickness is three different resonators. If we keep the resonators equally spaced, as it is shown in Supplementary Figure 2(c), the relation between the resonator impedances and the elements of the impedance matrix are

$$Z_1 = \frac{Z_0\det(Z)\sin(kl)}{j\det(Z)\cos(kl) + Z_0(Z_{22}+Z_{12})\sin(kl)} \tag{8}$$

$$Z_2 = \frac{Z_0^2 Z_{12}(\cos(2kl)-1)}{2\det(Z) + 2jZ_0Z_{12}\sin(2kl)} \tag{9}$$

$$Z_3 = \frac{Z_0\det(Z)\sin(kl)}{j\det(Z)\cos(kl) + Z_0(Z_{11}+Z_{12})\sin(kl)} \tag{10}$$

where $\det(Z) = Z_{11}Z_{22} - Z_{12}^2$. This configuration allows to independently control the three components

of the impedance matrix (Z_{11}, Z_{22}, and Z_{12}) with a fixed thickness of the cell by changing the physical dimensions of the resonators, i.e., the values of the impedances according to Supplementary Eqs. (8-10).

As an example, we can analyze the three-resonators model for the anomalous reflection scenario. Supplementary Figure 3(a) shows the values of the impedance matrix along one period when $\theta_\mathrm{i} = 0°$ and $\theta_\mathrm{t} = 80°$. This condition can be implemented using three resonators for a fixed separation between them $l = \lambda_0/4$ if when the resonators are described by the impedances represented in Supplementary Figure 3(b). As we can see from the high impedance values at some point of the period, some of the resonators are working in the resonant frequency.

Similar analysis can be applied to the four resonator approach and the transfer matrix can be expressed in terms of the four impedances:

$$\begin{aligned} M_{11} =& a^3 + 2abc + bcd + (a^2c + bc^2)Z_3 + Z_2(a^2c + acd + ac^2Z_3) + \\ & Z_1[a^2c + bc^2 + acd + cd^2 + (ac^2 + c^2d)Z_3 + Z_2(ac^2 + c^2d + c^2Z_3)] \end{aligned} \tag{11}$$

$$\begin{aligned} M_{22} =& abc + 2bcd + d^3 + (a^2c + bc^2 + acd + cd^2)Z_4 + \\ & Z_3[acd + +cd^2 + (ac^2 + c^2d)Z_4 + Z_3(c^2d + c^3Z_4)] \end{aligned} \tag{12}$$

$$M_{12} = a^2c + bc^2 + acd + cd^2 + (ac^2 + c^2d)Z_3 + Z_2(ac^2 + c^2d + c^3Z_3) \tag{13}$$

where $a = \cos(kl)$, $b = \mathrm{j}Z_0 \sin(kl)$, $c = \frac{\mathrm{j}}{Z_0} \sin(kl)$, $d = \cos(kl)$ are the components of the transfer matrix for a transmission line with length l. From these equations we can see that there are 3 equations and 4 unknowns, meaning that there will be infinite sets of solutions to the impedances. Therefor it is not trivial to define the geometry of the cells in theory, especially under certain geometric constraints. In light of this analysis, it it necessary that one needs optimization algorithms to help searching for a practical design for experimental implementations.

Supplementary Note 3. Retrieving impedance matrix in COMSOL

For the ease of implementation, the method we used to retrieve the impedance matrix in COMSOL is the same as the standard 4-microphone method for acoustic experiments with impedance tubes, whose setups are shown in Supplementary Figure 4. The positions of 4 microphones are x_1, x_2, x_3, x_4, respectively. By performing two measurements with different boundary conditions at the end of the tube, we can obtain four independent equations for determination of the four transfer matrix elements. Two different boundaries we used at the end of the tube are plane wave radiation (condition #1) and hard wall (condition #2). The pressure detected by these microphones under these two boundary conditions are noted as $p_m^{(n)}$ where m denotes the number of the microphone and n denotes the number of the boundary condition.They satisfy the condition:

$$\begin{bmatrix} e^{-jkx_1} & e^{jkx_1} \\ e^{-jkx_2} & e^{jkx_2} \end{bmatrix} \begin{bmatrix} A^{(1)} & A^{(2)} \\ B^{(1)} & B^{(2)} \end{bmatrix} = \begin{bmatrix} p_1^{(1)} & p_1^{(2)} \\ p_2^{(1)} & p_2^{(2)} \end{bmatrix} \tag{14}$$

Similarly,

$$\begin{bmatrix} e^{-jkx_3} & e^{jkx_3} \\ e^{-jkx_4} & e^{jkx_4} \end{bmatrix} \begin{bmatrix} C^{(1)} & C^{(2)} \\ D^{(1)} & D^{(2)} \end{bmatrix} = \begin{bmatrix} p_3^{(1)} & p_3^{(2)} \\ p_4^{(1)} & p_4^{(2)} \end{bmatrix} \tag{15}$$

With the measurement of $p_m^{(n)}$, all the ABCD in the matrices can be calculated. If the metasurface is located at x_0, then the pressure and velocity at the left side and right side can be written as:

$$\begin{bmatrix} p^{-(1)} & p^{-(2)} \\ v^{-(1)} & v^{-(2)} \end{bmatrix} = \begin{bmatrix} e^{-jkx_0} & e^{jkx_0} \\ e^{-jkx_0}/Z_0 & -e^{jkx_0}/Z_0 \end{bmatrix} \begin{bmatrix} A^{(1)} & A^{(2)} \\ B^{(1)} & B^{(2)} \end{bmatrix} \tag{16}$$

$$\begin{bmatrix} p^{+(1)} & p^{+(2)} \\ v^{+(1)} & v^{+(2)} \end{bmatrix} = \begin{bmatrix} e^{-jkx_0} & e^{jkx_0} \\ e^{-jkx_0}/Z_0 & -e^{jkx_0}/Z_0 \end{bmatrix} \begin{bmatrix} C^{(1)} & C^{(2)} \\ D^{(1)} & D^{(2)} \end{bmatrix} \tag{17}$$

Therefore, the transfer matrix of the measured unit cell can be calculated as

$$T = \begin{bmatrix} p^{+(1)} & p^{+(2)} \\ v^{+(1)} & v^{+(2)} \end{bmatrix} \begin{bmatrix} p^{-(1)} & p^{-(2)} \\ v^{-(1)} & v^{-(2)} \end{bmatrix}^{-1} \tag{18}$$

Hence the impedance matrix can is calculated as

$$Z = \begin{bmatrix} -\frac{T_{22}}{T21} & -\frac{1}{T21} \\ \frac{T_{12}T_{21}-T_{11}T_{22}}{T21} & -\frac{T_{11}}{T21} \end{bmatrix} \tag{19}$$

Supplementary Note 4. Numerical simulations when loss is considered

Although the structure is designed with lossless assumption, the performance is also confirmed in simulation by considering viscous loss since it is the inherent loss of the structure which is inevitable in the experiments. Loss in the air is modeled by the viscous fluid model in the Pressure Acoustic Module in COMSOL, with dynamic viscosity of 1.82×10^{-5}Pa and bulk viscosity of 5.46×10^{-2}Pa. The simulated fields are shown in Supplementary Figure 4. The simulations show that for the bianisotropic design, the amplitude of the transmission coefficient decays to $T = 1.31$ indicating that 85% of the energy is transmitted to the desired direction. This value is still higher than lossless discretized GSL-based designs. This confirms that the performance of the designed metasurface will not be severely influenced when loss is considered. This is because the proposed metasurface is not resonant based, and all the resonators are designed so that the operating frequency is far off resonance [see Supplementary Note 7].

Supplementary Note 5. Comparison between transfer matrix method and numerical simulations

To evaluate the performance of the transfer matrix, we compare the transmission and reflection characteristics of a cell both analytically and numerically. The dimensions of the cell is the same as Fig.2 in the main text, i.e., $w = 12$ mm, $h_2 = 1.5$ mm, $w_2 = 1$ mm, $h_1 = 1$ mm $w_1 = 4$ mm, $w_\mathrm{a} = 6$ mm, $w_\mathrm{b} = 5$ mm, $w_\mathrm{c} = 4$ mm, and $w_\mathrm{d} = 3$ mm. The lines represent the results from the transfer matrix method and the markers represent numerical simulations from COMSOL. Good agreement can be observed and the results confirm the biansiotropic nature of the structure proposed, as the reflected phase from opposite directions are different.

Supplementary Note 6. Convergence of the optimization process in each design

To show the convergence of the optimization process, the evolution of the cost function with the evolution of the algorithm for the first cell of the 60° case is shown in Supplementary Figure 7 as an example. For cleanness, here we only showed 10 evolution results.

Supplementary Note 7. Design parameters of the scattering-free anomalous refractive metasurfaces

This section details the physical dimensions of the final designs for the anomalous refractive metasurfaces when $\theta_{\mathrm{i}} = 0°$ and $\theta_{\mathrm{t}} = 60°$ (Supplementary Table 1), $\theta_{\mathrm{t}} = 70°$ (Supplementary Table 2), and $\theta_{\mathrm{t}} = 80°$ (Supplementary Table 3).

Supplementary Note 8. The necessity of using four resonators

From the discussion in Supplementary Note 2, the minimum requirement to achieve bianisotropy is to use three resonators. However, as we will show here, the use of only three resonators will have certain limitations in practical implementations. For example, it will induce larger error, greater instability/sensitivity to the geometry and thus fabrication error, closer to resonant frequencies, and is therefore not employed in our design. To illustrate the necessity of using four resonators, we choose unit #1 of the 60° deflection case as an example. To replace the four-resonator cell with the three-resonator cell, the width of the cell ($w = 12$ mm), thickness of the shell ($h_1 = 1$ mm), width of the neck ($h_2 = 1.5$ mm) and length of the cell (50 mm) is kept the same while the height of the channel (w_1) and the cavities ($w_{a,b,c}$) are set as variable. We run a set of Genetic Algorithm (GA) by following the same procedure with the four-resonator design to find the optimal geometry produced by using three resonators. However, the results will not converge and an acceptable design (within 20% error) can not be found. This is because the individual resonators cannot operate near the resonance under the given geometry (same as four-resonator designs), and without accessing the extreme values near the resonance, the resulting whole impedance matrix cannot provide the bianisotropic response required by the theory. To release this condition, we reduce the width of the h_2 neck to 0.6 mm, so that the resonators can operate near resonance within the range of the cavity height (0 mm to 9mm). Moreover, the height of the necks of each resonator is also set to be variables so that the resulting geometry would provide more degrees of freedom. By relaxing these constraints and running GA, a design is found with a 4.47% cost. The parameters are $w_1 = 3.2$ mm, $w_a = 4.7$ mm, $w_b = 4.5$ mm, $w_c = 3.6$ mm, with the height of the necks being 0.63 mm, 0.87 mm and 1.15 mm, respectively. Supplementary Figure 8 shows the total acoustic field of the three-resonator cell compared with the four-resonator cell.

It can be seen that the three-resonator cell can generally produce the same response compared with the four-resonator cell. However, one should bear in mind that such a unit cell based on three resonators is achieved by releasing some of the geometrical constraints such as the height of the necks. This will inevitably make the designing process more complicated, and the narrow neck width and varying neck height will also

pose challenges to fabrication and may result in less stability and repeatability. Moreover, the small features would also make the whole structure very sensitive to fabrication errors.

In addition, we also calculate the resonance frequencies of each individual resonator in the three-resonator design, the resonance frequencies are 3.96 kHz, 3.85 kHz, and 3.97 kHz, respectively, which are all much closer to the designed operation frequency (3.0 kHz) compared to the four-resonator design. This confirms that the use of only three resonators will make the resonators work near their resonance, which will in turn increase the loss in real implementation. The use of four resonators, on the other hand, will effectively reduce the requirement for each individual resonator, making them work away from resonance.
